# Genotyping of *Anopheles* mosquito blood meals reveals nonrandom human host selection: implications for human-to-mosquito *Plasmodium falciparum* transmission

**DOI:** 10.1186/s12936-023-04541-2

**Published:** 2023-04-07

**Authors:** Rex B. Mbewe, John B. Keven, Charles Mangani, Mark L. Wilson, Themba Mzilahowa, Don P. Mathanga, Clarissa Valim, Miriam K. Laufer, Edward D. Walker, Lauren M. Cohee

**Affiliations:** 1 Department of Entomology, Michigan State University, East Lansing, MI USA; 2 Department of Physics and Biochemical Sciences, Malawi University of Business and Applied Sciences, Blantyre, Malawi; 3 Department of Public Health, College of Health Sciences, University of California-Irvine, Irvine, CA USA; 4 Malaria Alert Center, Kamuzu University of Health Sciences, Blantyre, Malawi; 5 Department of Epidemiology, School of Public Health, University of Michigan, Ann Arbor, MI USA; 6 Department of Global Health, Boston University School of Public Health, Boston, MA USA; 7 Malaria Research Program, Center for Vaccine Development and Global Health, University of Maryland School of Medicine, Baltimore, MD USA; 8 Department of Microbiology and Molecular Genetics, Michigan State University, East Lansing, MI USA

**Keywords:** Malaria parasite transmission, Microsatellites, Vector-borne disease prevention, Age-specific risk, Human reservoirs of infection

## Abstract

**Background:**

Control of malaria parasite transmission can be enhanced by understanding which human demographic groups serve as the infectious reservoirs. Because vector biting can be heterogeneous, some infected individuals may contribute more to human-to-mosquito transmission than others. Infection prevalence peaks in school-age children, but it is not known how often they are fed upon. Genotypic profiling of human blood permits identification of individual humans who were bitten. The present investigation used this method to estimate which human demographic groups were most responsible for transmitting malaria parasites to *Anopheles* mosquitoes. It was hypothesized that school-age children contribute more than other demographic groups to human-to-mosquito malaria transmission.

**Methods:**

In a region of moderate-to-high malaria incidence in southeastern Malawi, randomly selected households were surveyed to collect human demographic information and blood samples. Blood-fed, female *Anopheles* mosquitoes were sampled indoors from the same houses. Genomic DNA from human blood samples and mosquito blood meals of human origin was genotyped using 24 microsatellite loci. The resultant genotypes were matched to identify which individual humans were sources of blood meals. In addition, *Plasmodium falciparum* DNA in mosquito abdomens was detected with polymerase chain reaction. The combined results were used to identify which humans were most frequently bitten, and the *P. falciparum* infection prevalence in mosquitoes that resulted from these blood meals.

**Results:**

*Anopheles* females selected human hosts non-randomly and fed on more than one human in 9% of the blood meals. Few humans contributed most of the blood meals to the *Anopheles* vector population. Children ≤ 5 years old were under-represented in mosquito blood meals while older males (31–75 years old) were over-represented. However, the largest number of malaria-infected blood meals was from school age children (6–15 years old).

**Conclusions:**

The results support the hypothesis that humans aged 6–15 years are the most important demographic group contributing to the transmission of *P. falciparum* to the *Anopheles* mosquito vectors. This conclusion suggests that malaria control and prevention programmes should enhance efforts targeting school-age children and males.

**Supplementary Information:**

The online version contains supplementary material available at 10.1186/s12936-023-04541-2.

## Background

Differential and biased feeding by vectors on particular human demographic groups can have profound impacts on malaria epidemiology and may permit *Plasmodium* transmission to persist even where control measures are in place [1–7]. One approach to quantifying patterns of feeding on human hosts of different ages and sexes is with blood meal analysis [8]. Blood meal analysis serves to estimate the human blood index (proportion of hosts that are humans) as well as to estimate the proportion of mosquito infections originating from different demographic groups in the human population [3, 9, 10].

In Malawi, prevalence of *Plasmodium falciparum* infection is highest in school-age children (SAC) [11–13]. Furthermore, infections in this age group are more likely to contain gametocytes, the sexual stage of the parasite required for human-to-mosquito transmission [14], suggesting that SAC may be the most important reservoir of infection [12, 15]. However, to quantify the contribution of this age group to transmission requires determining the frequency with which vectors take blood meals from this age group in comparison to other age groups.

Genetic profiling of human blood meals has been applied in various transmission settings to identify the most frequently bitten population group, assess the frequency distribution of mosquito biting on individuals, and estimate the proportions of multiple blood meals on different human hosts [3, 9, 10]. It utilizes microsatellite genetic markers, or short tandem repeats (STRs) of nucleotides, which occur at thousands of Mendelian loci in the human genome with multiple alleles operating at each locus [16]. The resultant genetic variation provided by the combination of numerous loci and multiple alleles provides for various applications in research such as studies of population genetic structure [17] and in forensic sciences [18, 19].

Previous studies prior to widespread access to bed nets suggested that, based largely on differences in body surface area, a greater proportion of blood meals are taken from adults and older children than young children and infants [20, 21]. SAC are the least likely age group to report sleeping under bed nets in Malawi and elsewhere, suggesting they may be more available to malaria vector feeding [13, 22, 23]. Furthermore, *Plasmodium-*infected people may be more attractive to mosquito vectors than uninfected people [24, 25].

To assess the relative contribution of human age and sex demographic groups to mosquito feeding and parasite transmission, this study compared genotypes of human blood from mosquito blood meals to the blood samples taken from humans sleeping in households where the mosquitoes were collected.

## Methods

### Study site

Mosquito and human samples were collected as part of a household-based cohort study conducted in two districts (Machinga and Balaka) in southeastern Malawi where incidence of malaria is moderate to high throughout the year [26]. According to the Malawi National Statistical Office census report for 2018, Machinga district had a total population of 735,438 with a population density of 205/km^2^ on a 3582 sq. km land area, while Balaka district had a total population of 438,378 with a population density of 205/km^2^ on a 2142 sq.km land area. The residents of the area are mostly subsistence farmers whilst others ply trade in market centers. Malaria transmission is highest in areas of high temperature and seasonal rainfall, from October through April [27]. Long-lasting insecticidal nets were distributed via a mass campaign in September–November 2018. In the study sites 91% of households reported owning at least one net and 74% of residents reported sleeping under a net (Malawi ICEMR, unpublished data).

### Household surveys

**Fig. 1:**
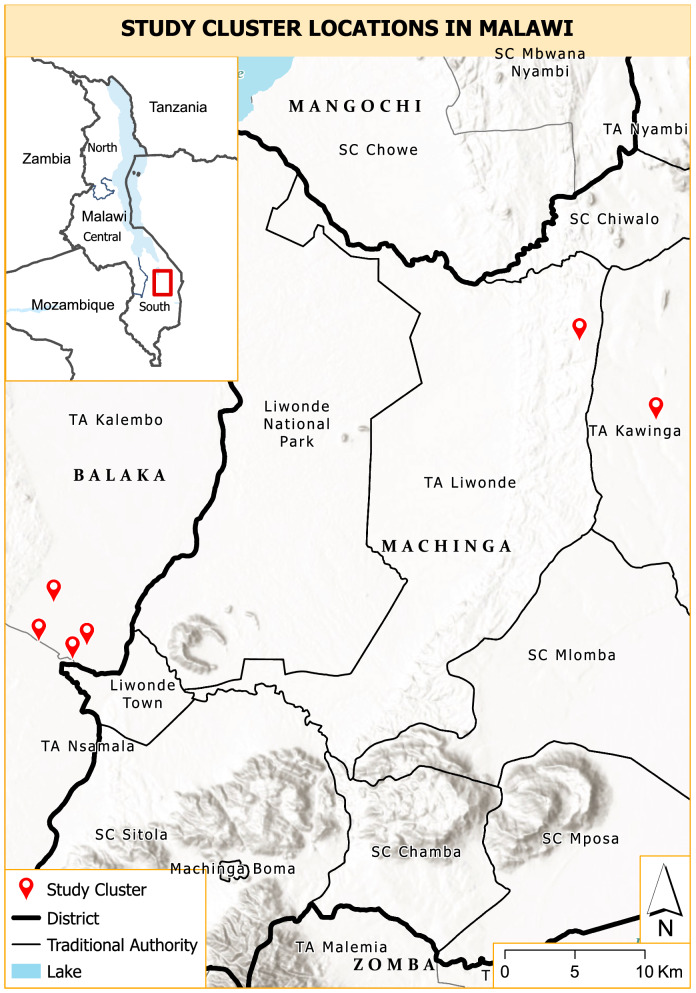
Map of Malawi showing the study site and sampling cluster locations

Monthly surveys of a household-based cohort were conducted to collect demographic and malariometric data in 46 households in six clusters. Clusters were created as part of a larger study (detailed methods not yet published, [28]) by randomly selecting an index household and inviting 5–7 nearest neighbours to also enroll in the study (Fig. 1). A questionnaire designed to gather information on the number of people per household, and the name, sex and date-of-birth of each household member was administered to each household head who consented to participate. Blood was collected by finger prick from consented household members, dried on Whatman filter paper, and stored under cool condition.

Indoor-resting, female *Anopheles* mosquitoes were sampled from the sleeping spaces of the enrolled households using resting collections, pyrethrum spray catches, and CDC miniature light traps. The households were sampled one night every 2 weeks from February to April of 2020, yielding 4 to 6 nights of mosquito collection per household. The individuals who slept in the house the night prior to sample collection were considered to be at risk. The total nights at risk was calculated as the sum of the number of collection nights in which each participant was recorded as having slept in the household.

### DNA extraction

Each blood-fed *Anopheles* mosquito was bisected into the head attached to the thorax (hereafter, “head-thorax”), and the abdomen, using sterile technique. Genomic DNA was extracted from the mosquito abdomens and from human blood on filter papers using the DNeasy^®^ Blood & Tissue Kit (Cat. No. 69582; Qiagen, Valencia, CA, USA).

### Anopheles species identification

Mosquitoes morphologically identified as members of either the *Anopheles gambiae* complex or *Anopheles funestus* group [29] and subjected to polymerase chain reaction (qPCR) methods described elsewhere [26] to determine the *Anopheles* species.

### Plasmodium falciparum detection

Presence of *P. falciparum* parasites in human blood samples and mosquito abdomens was tested using a qPCR method targeting the 18S rRNA gene [3].

### Identification of human blood meals

DNA samples obtained from blood-fed mosquito abdomens were subjected to a qPCR method containing primers (forward 5′-GGCCTGTTCCTCCCTTATTT-3′, reverse 5′-TACACAGGGCTTCCGAGT-3′) and probe (FAM-ATGGAGTCTGTGTTCCCTGTGACC-QSY) that detects intron 1 of the human tyrosine hydroxylase gene [26, 30].

### Microsatellite genotyping and profile matching

Human blood from mosquito blood meals and blood samples drawn from people were subjected to a microsatellite genotyping method [3]. The method involved PCR-amplification and genotyping of 23 well-characterized microsatellite loci, plus a sex-linked gene (amelogenin) locus to identify the sex of an individual [3, 31] (also see Supplementary Text). Amplification was performed using a validated, commercially available multiplex PCR kit (Promega, PowerPlex Fusion System; catalog no. DC2402) that contained locus-specific fluorescent-labelled primers [31]. PCR products were analysed by capillary electrophoresis (ABI 3730 Genetic Analyzer, Applied Biosystems, Foster City, CA) with LIZ 500 (Applied Biosystems, Foster City, CA) as the internal size standard. GeneMapper software version 4.1 (Applied Biosystems) was used to visualize the allelic sizes of all the loci in each sample (Additional file 1: Fig. S1). For each sample (i.e., human-fed mosquito or human blood), all observed allele sizes at each locus were listed and together served as the genetic profile of the person (Additional file 3: Table S2 and Additional file 4: Table S3). While different mosquitoes that fed on the same individual had the same genetic profile, each study participant had a unique genetic profile, except for monozygotic twins, which are extremely rare in the study communities. The genetic profile of a mosquito blood meal from a household was compared to the genetic profile of all members of that household, and to the entire human survey, to determine which person was fed on by the mosquito.

### Data analysis

Genotyping data were analysed as previously described [3] using R software (Version 4.0.2; https://www.R-project.org/), and an R script that compared the genotype of each locus in a genetic profile to its corresponding locus in other genetic profiles. The similarity of the two genetic profiles was expressed as the proportion of identical alleles at all loci times 100. For example, 19 random loci with identical genotypes divided by 24 total loci multiplied by 100 returns a 79% profile similarity. For mosquitoes whose blood meals originated from the same person, the profile similarity would be 100%. However, similarities less than 100%, for blood meal samples obtained from the same person could result from the so-called “allele dropout” effect which is a failure of one or more alleles at a locus to be detected at all because of low DNA concentration or quality [32]. To account for this possible source of error, and to minimize its effect in obtaining a reliable comparison of genotypes between pairs of blood meal samples and between blood meals and human blood samples, a value less than 100% must be used as the criterion for establishing a match from same source. To determine this value empirically with confidence, a pairwise matching analysis of genotypes was performed on the genetic profiles of every individual human in the study sites who provided a blood sample. That is, for each person, his/her genetic profile was compared with the genetic profile of every other individual in the study site and the similarity value of each pairwise match was recorded. It is worth noting that unlike the blood meal samples, which are prone to allele drop out due to low DNA quantity (small blood meal volume) or quality (partially digested blood), the DNA isolated from the human blood samples had higher quantity and quality and produced quality genetic profiles that warranted their use in the pairwise analysis for the match criterion. The pairwise comparison process generated [n(n-1)]/2 similarity values for profile matches, where n is the total number of humans sampled at a site. A similarity value higher than the highest value in the pairwise match output (excluding match results for monozygotic twins, which would be 100%) was then established as the criterion below which two genetic profiles were considered to be different [3].

Blood meals from multiple humans that could not be matched to any single genotype, owing to the presence of more than two alleles at many loci, were excluded from any matching analyses. After excluding the mixed blood meal profiles, genetic profiles of single-human blood meals were compared to each other and to the number of different human individuals in the blood meal sample, to determine the number of unique genetic profiles present in that sample. The frequency of occurrence of each unique genetic profile in the blood meal sample represented the number of mosquitoes that fed on each individual human. These data were used to construct frequency distribution histograms that relate the number of different human individuals (y-axis) to the number of blood meals they provided (x-axis). The observed frequency distributions were fitted to zero-truncated Poisson and zero-truncated negative binomial frequency distribution models using the functions zerotrunc and rootogram of the R package countreg [33]. The fit of the two distributions was compared by *x*^2^ test to determine if selection of humans by mosquitoes was random (Poisson) or non-random and aggregated (negative binomial). Genotypes of human blood-fed mosquitoes (blood meals) were compared to each other, and to genotypes of human individuals.

Genetic profiles of blood meals were also compared to genetic profiles of individual humans. Because a sex-specific marker was included in the genotyping process, the sex of the source of all human blood meals could be determined without the need to match the blood meal profiles to those of the study participants. However, the age of the source of human blood meals could only be determined for blood meals that matched study participants. Age was analysed categorically as ≤ 5, 6–15, 16–30 and 31–75 years old. The proportion of different sex and age groups observed in the mosquito blood meals was compared with their expected proportion (the proportion of individuals of that group in the household survey which represented the population at risk) using two-tailed binomial tests. Blood meals from multiple humans that could not be matched to any single genotype, owing to the presence of more than two alleles at many loci, were excluded. Proportions of infected individuals across age categories were compared using contingency tables and *x*^2^ test for trend. Logistic regression analyses were used to confirm associations, but data are presented in tables for clearer interpretation.

## Results

**Fig. 2:**
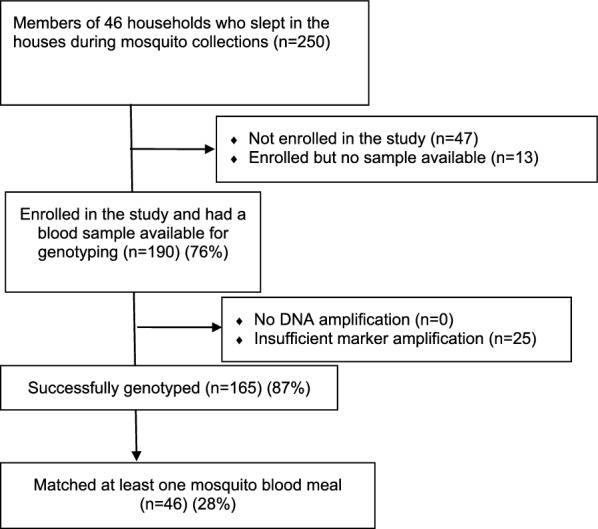
Flow chart summarizing the number of humans enrolled in the study, and blood samples collected, processed, and compared with blood in *Anopheles* mosquito abdomens

**Fig. 3:**
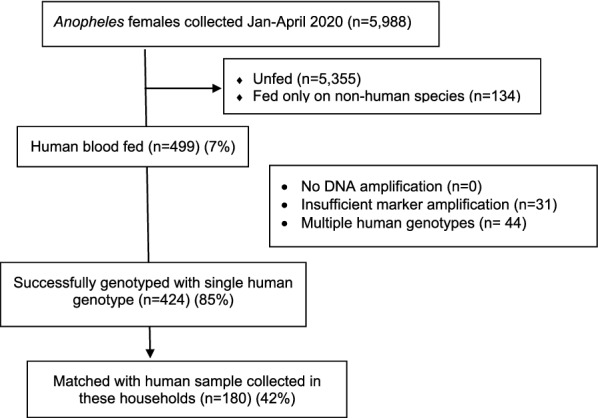
Flow chart summarizing results of *Anopheles* mosquito blood meals from collections in study households

A total of 5,988 *Anopheles* spp. mosquitoes were collected from repeated sampling of 46 households (Figs. 2 and 3). Among these, 633 mosquitoes were blood-fed with 499 having fed upon humans (human blood feeding index 78.8%). Two-hundred and fifty people slept in households in which these mosquitoes were collected. Samples were available from 190 (76%) of these people.

### Similarity of human genetic profiles

Quality genetic profiles were successfully generated from 165 (87%) of the 190 sampled human individuals (Fig. 2). The genetic profiles of the remaining 13% of people were of poor quality and thus excluded from subsequent analyses. A comparison was made of each genetic profile with all others, including between siblings, to determine the degree of similarity, resulting in 29,161 pairwise genotype matches. There were 20 unique similarity values ranging from 0 to 79%, each with a varying frequency (probability) of occurrence (Additional file 2: Table S1). The highest similarity value of 79% (i.e. 19 of 24 matched loci) had a probability of occurrence of 10^–5^ (Additional file 2: Table S1), which is extremely low and there were only two such matches. This result means that no two non-monozygotic individuals including siblings in the study sites had a pairwise similarity value greater than 79%. Thus, this value was chosen as the criterion above which two blood meal genetic profiles were considered to be from the same human source. Conversely, samples with pairwise values ≤ 79% were considered to be from different human sources. It is worth noting here that the absence of 100% match value in the data indicates that the study participants (not the entire population) did not include monozygotic twins. Although monozygotic twins could be present in the population and the blood meal profiles from the two different individuals could be confused for a single person, the effect of this error on the data is negligible given the rarity of monozygotic twins in a population.

### Genotyped human blood meals

Of 499 *Anopheles* spp. mosquitoes with blood meals containing human blood, 468 (93.8%) yielded analyzable human genotypes (Fig. 3). Of these, 424 (90.6%) were found to contain a single genotype whilst 44 (9.4%) contained mixed blood meals from two or more humans and could not be genotyped to identify unique individuals, and hence were not used in the matching process. The 424 female *Anopheles* mosquitoes containing blood meals with a single genotype included *Anopheles arabiensis* (187, 44.1%), *An. gambiae stricto *sensu (66, 15.6%), *An. funestus* (150, 35.4%), *Anopheles parensis* (1, 0.2%), *Anopheles vaneedeni* (1, 0.2%) and unidentified *Anopheles* species (19, 4.5%). When the genotypes of 424 single-human blood meals were compared with the 165 human blood genotypes of the household residents, 180/424 (42.5%) blood meals matched to 46/165 (27.8%) of the study participants. The other 244/424 (57.5%) blood meals contained profiles from 119 unique humans whose genotype did not match the genotype of any of the household residents enrolled in the study and represented in the sample.

### Frequency distribution of human host selection

**Fig. 4:**
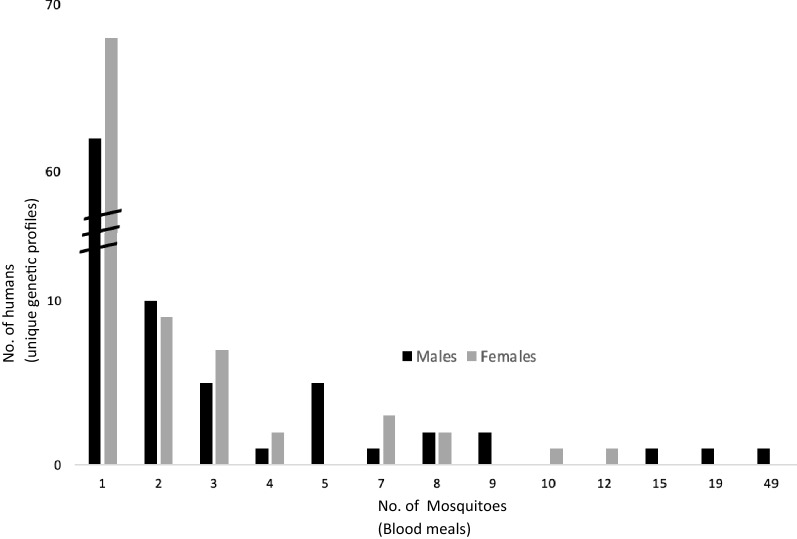
Frequency distribution of number of blood meals on 424 individual male (n = 256) and female (n = 168) humans taken by *Anopheles* mosquitoes sampled in communities of southeastern Malawi, showing the number of mosquito bites per person by sex

The number of blood meals taken from individual people ranged from 1 to 49. The frequency distribution of genotyped blood meals taken from individual humans showed that some people were more frequently fed upon than others. More than 60 individual humans contributed only one blood meal, each to a single mosquito, while three male humans each individually contributed blood meals to 15, 19, and 49 mosquitoes, respectively (Fig. 4).

The frequency distribution fit the negative binomial distribution well (*x*^2^ = 9.4306, df = 9, *P* = 0.3985), but did not fit the Poisson distribution (*x*^2^ = 82.396, df = 6, *P* =  < 0.0001), indicating a nonrandom and aggregated (or clumped) pattern of human host selection.

### Mosquito feeding on human hosts by sex and age

Categorization of all genotyped blood meals into male and female human blood source and comparing the data to the proportion of males and females in the population at risk revealed that males contributed a significantly higher proportion of blood meals than females (Table 1). Among successfully matched blood meals from which the age of the human blood source was known, children ≤ 5 years of age were under-represented in mosquito blood meals compared to their availability in the population at risk of being bitten. School-aged children (SAC, 6–15 years old) and young adults (16–30 years old) were represented in mosquito blood meals in equal proportion to their presence in the population. However, older adults (31–75 years old) were overrepresented in mosquito blood meals. Comparisons by sex within each age group revealed that males are the primary source of blood meals in the older age groups. Regression analysis including the interaction of sex and age group confirmed these associations (Table 1).

**Table 1:** Comparison of population at risk of being bitten (expected proportions) successfully matched genotypes of human individuals blood meals sources of different sex and age groups (observed proportions)

Human host		No. At-risk nights (Expected % of blood meals)	No. genotyped blood meals (Observed % of blood meals)	Expected vs. Observed p-value	Sex	No. At-risk nights (Expected % of blood meals)	No. genotyped blood meals (Observed % of blood meals)	Expected vs. Observed p-value
Sex^a^	Male	468 (44.7%)	256 (60.4%)	**0.002**	–	–	–	–
	Female	579 (55.5%)	168 (39.6%)	**0.001**	–	–	–	–
Age^a^ (years)	≤ 5	192 (24.9%)	14 (7.8%)	** < 0.0001**	Male	85 (11.04%)	5 (2.78%)	**< 0.001**
					Female	107 (13.9%)	9 (5.00%)	**0.003**
	6–15	278 (36.1%)	62 (34.4%)	0.68	Male	164 (21.30%)	27 (15.00%)	0.12
					Female	114 (14.81%)	35 (19.44%)	0.19
	16–30	117 (15.2%)	26 (14.4%)	0.81	Male	39 (5.06)	19 (10.56%)	**0.010**
					Female	78 (10.13%)	7 (3.89%)	**0.014**
	31–75	183 (23.8%)	78 (43.3%)	** < 0.0001**	Male	49 (6.40%)	63 (35.00%)	** < 0.0001**
					Female	134 (17.40%)	15 (8.33%)	**0.008**

^a^Total numbers for sex and age differs because sex is available for every human genotype in blood meals, whereas age requires matching to an enrolled participant. No. of At-risk nights was calculated as the sum of the number of collection nights participants in each demographic category slept in the household

P-value in **Bold** indicates that this demographic group contributed more or less blood meals than expected

Males contributed more blood meals than expected in proportion to their availability in the population. This was especially true for age group 31–75 years old. Logistic regression analysis revealed the same associations

### Plasmodium falciparum infection in Anopheles blood meals

*Plasmodium falciparum* was detected in 151 (36%) of the 424 genotyped blood meals of *Anopheles* mosquitoes. The proportions of infections were similar for blood meals taken from females (n = 59, 35%) and males (n = 92, 36%) (Table 2), and results were similar if analyses were restricted to blood meals that matched study participants. Of the successfully genotyped and matched blood meals (n = 180), the proportion of *Anopheles* blood meals positive for *P. falciparum* decreased as age of the human source of the blood meal increased (p < 0.0001, test for trend). While the highest proportion of infected blood meals was found in blood meals obtained from young children (≤ 5 years old), the largest number of infected blood meals was obtained from SAC (6–15 years old). Data were too sparse to make more meaningful inferences on the distribution of infection by sex within age group (Additional file 3: Table S2). Thus, overall, SAC contributed the most infection to mosquitoes, thereby further perpetuating transmission (Table 2).

**Table 2:** Comparison of blood meals sources containing *P. falciparum* parasites by sex and age of the human genotype detected in the blood meal

Human host		Infected blood meal^a^	Uninfected blood meal^a^	p-value
Sex	Male	92 (0.36)	164 (0.64)	0.86
	Female	59 (0.35)	109 (0.65)	
Age (years)	≤ 5	8 (0.57)	6 (0.43)	< 0.0001^b^
	6–15	26 (0.42)	36 (0.58)	
	16–30	3 (0.12)	23 (0.88)	
	31–75	12 (0.15)	66 (0.85)	

^a^Totals differ because sex is available for every human genotype in blood meals, whereas age requires matching to an enrolled participant

^b^Chi-square trend analysis

## Discussion

This study shows that in the setting of current net ownership and use patterns, *Anopheles* mosquito feeding on humans was non-random, with more blood meals taken from older males. However, school-age and under-five children transmitted more *P. falciparum* infections to mosquitoes, both relatively and absolutely. These results underscore the importance of understanding both vector feeding behaviour and human infectiousness in designing targeted interventions for malaria control. Although they were more frequently bitten, adult males may not be the most important target for malaria control through decreased vector-human contact. Instead, reduced population-level *P. falciparum* transmission might be better achieved by decreasing mosquito feeding on the more infectious ≤ 5 year olds and SAC.

The finding that human-host blood feeding was non-random and aggregated has also been reported in other studies using genetic profiling of *Anopheles*, *Culex*, and *Aedes* blood meals [34–37]. Non-random feeding is likely due to differences in mosquito access to individual humans or houses as a result of differences in the distance from larval habitat, use of LLINs and repellents, late night activity, cooking location, and/or house construction that impedes mosquito entry [38, 39]. Also, humans are more likely to encounter host-seeking *Anopheles* where these mosquitoes are abundant, and during activities that may differ according to people's age and sex [40, 41]. Thus, human behaviour likely plays an important role in determining which humans are fed upon.

The observation that mosquitoes disproportionately acquired blood meals from older people aligns with results of studies conducted elsewhere. In Papua New Guinea, Kenya, Tanzania and Burkina Faso, *Anopheles* mosquitoes were generally observed to obtain more blood meals from males and older age groups compared to the youngest ages [3, 10, 42]. The youngest age groups and women of childbearing age could be receiving fewer mosquito bites than older males because they are more likely to be under the protection of bed nets.

Blood feeding on multiple humans during the same gonotrophic cycle was common and has several implications for malaria epidemiology. Arthropod vectors that take multiple human blood meals generally will increase the vectorial capacity of the vector population by increasing the chances of both acquiring and transmitting the pathogen [43]. More partial blood meals per gonotrophic cycle increase transmission above that resulting from single feedings and the feeding of *Anopheles* mosquitoes on two human hosts per gonotrophic cycle increases both* R*_*0*_ and *VC* by two-fold or greater [44]. The present study showed that 1 in 10 mosquito blood meals were taken from multiple individuals. This finding is consistent with other studies conducted in Papua New Guinea, Burkina Faso and Kenya, where the proportions of multiple blood meals ranged from 6 to 15%, 11–18%, 12–16%, and 0–14% [3, 10, 42, 45], respectively. Future studies should incorporate quantification of multiple feeding on malaria parasite transmission dynamics and intervention policy.

*Plasmodium* infection prevalence differed among age groups. The present study showed that blood meals from SAC had higher *P. falciparum* infection prevalence than the other age groups and that no significant differences were observed between male and females. Keven et al*.* [3] found that in one Papua New Guinea village, males and age group 15–30 years old had a higher probability of infection than females and other age groups. The consistency in these two studies is the observation that some human population groups have a higher likelihood of malaria parasite infection than others. As such, these population groups would be more responsible for malaria parasite transmission to mosquitoes.

An important strength of the present study was the use of 24 loci for genetic profiling, which increased the sensitivity of discrimination as compared to previous studies that analysed fewer markers [10, 34, 35, 37, 42, 46, 47]. Using 24 microsatellite loci not only increased power to discriminate between genetically related individuals, but also decreased the possibility of error due to allele dropouts [3, 48]. Thus, the assessment was more robust against matching errors. Further, the current study is among the few that have attempted to detect infection in the mosquitoes based on the sex and age of the human source of blood meal and inferring human-to-mosquito parasite transmission.

More than half of analysed blood meals did not match the genotype of an enrolled participant, which represents a limitation for analyses such as age that required a match. This might be because some indoor resting mosquitoes took their blood meal outdoors from non-enrolled neighbours, household visitors, or from non-participating household members. Harrington et al*.* [36] reported that most of the blood meals taken by *Aedes aegypti* in Thailand villages came from non-household members, a finding attributed to peddlers, visitors, and passersby in the community during the daytime hours, when *Ae. aegypti* most often seeks hosts. In the current study, the number of non-resident visitors in or near the study households was not assessed either during the day or the night/early morning when *Anopheles* mosquitoes seek hosts. However, the genotyped blood meal data suggest that such human movement was considerable. Also, the few blood meals that were determined to be from multiple humans were not analysed. Finally, the analyses included an assumption that the population distribution in other households was similar to study households. Future studies will be stronger if they maximize the genotype matches by simultaneously sampling for humans and mosquitoes in each household, obtaining blood samples from visitors, and genotypically identifying unique individuals in blood meals from multiple human sources.

This study analysed *Plasmodium* infections detected in the mosquito abdomen to infer infection transmission from humans to mosquitoes. While *P. falciparum* parasites in the abdomen do not always produce infectious sporozoites, their presence in the abdomen serves as a starting point for future parasite transmission (mosquitoes becoming infectious). Others studies have shown that a high proportion of infections in SAC contain gametocytes, the parasite stage required for transmission, [12, 14, 49] and that infections in this age group are highly transmissible in mosquito feeding assays [10, 50, 51]. The current study further suggests that the school-age group could be the primary reservoir and main driver of *Plasmodium* infection into mosquitoes.

Non-random vector feeding has important epidemiological implications. Several simulation modeling studies have shown that non-random human blood feeding results in a greater Basic Reproductive number (*R*_*o*_), which increases the probability that transmission persists [1, 5, 45], even in the presence of malaria control programmes such as distribution of bed nets [4]. Therefore, targeted interventions focusing on those human demographic groups that disproportionately serve as *Plasmodium* infection reservoirs should enhance reduction of malaria parasite transmission on top of uniform interventions.

## Conclusion

The *Anopheles* mosquito population in the study area of southern Malawi exhibited non-random human host feeding, with males and the older adults being most frequently bitten, in the setting of high levels of net ownership and moderate levels of reported use. However, the prevalence of *P. falciparum* infection in mosquitoes was highest when the blood meal source was from ≤ 5 years old and SAC. Because younger children were the source of fewer blood meals, SAC are the source of the majority of mosquito infections. These findings are crucial for efforts to focus malaria control and prevention at the demographic groups that are currently most important to maintaining transmission, specifically SAC.

## Supplementary Information

**Additional file 1: Figure S1.** Example of output for visualization of allelic sizes on each loci for creating genetic profiles of each individual sample.

**Additional file 2: Table S1.** Percent of loci matched, pairwise testing matches and probability of having similar genetic makeup among 190 sampled human individuals (29,161 pairwise comparisons) from 46 different households.

**Additional file 3: Table S2.** Demographic age groups separated by sex showing their P. falciparum infection proportions.

**Additional file 4: Table S3.** Example of profiles generated from Human bloodspot samples.

**Additional file 5: Table S4.** Example of profiles generated from Mosquito blood meal samples.

## Data Availability

The data will be available upon request made to the corresponding author.

## References

[1] Dye C, Hasibeder G (1986). Population dynamics of mosquito-borne disease: effects of flies which bite some people more frequently than others. Trans R Soc Trop Med Hyg.

[2] Hasibeder G, Dye C (1988). Population dynamics of mosquito-borne disease: persistence in a completely heterogeneous environment. Theor Popul Biol.

[3] Keven JB, Katusele M, Vinit R, Rodríguez-Rodríguez D, Hetzel MW, Robinson LJ (2021). Nonrandom selection and multiple blood feeding of human hosts by *Anopheles* vectors: implications for malaria transmission in Papua New Guinea. Am J Trop Med Hyg.

[4] Smith DL, McKenzie FE, Snow RW, Hay SI (2007). Revisiting the basic reproductive number for malaria and its implications for malaria control. PLoS Biol.

[5] Woolhouse MEJ, Dye C, Etard J-F, Smith T, Charlwood JD, Garnett GP (1997). Heterogeneities in the transmission of infectious agents: implications for the design of control programs. Proc Natl Acad Sci USA.

[6] Melgarejo-Colmenares K, Cardo MV, Vezzani D (2022). Blood feeding habits of mosquitoes: hardly a bite in South America. Parasitol Res.

[7] Jeyaprakasam NK, Low VL, Liew JWK, Pramasivan S, Wan-Sulaiman W-Y, Saeung A (2022). Blood meal analysis of *Anopheles* vectors of simian malaria based on laboratory and field studies. Sci Rep.

[8] Borland EM, Kading RC (2021). Modernizing the toolkit for arthropod bloodmeal identification. Insects.

[9] Escobar D, Ascencio K, Ortiz A, Palma A, Sánchez A, Fontecha G (2020). Blood meal sources of Anopheles spp. in malariaeEndemic areas of Honduras. Insects.

[10] Gonçalves BP, Kapulu MC, Sawa P, Guelbéogo WM, Tiono AB, Grignard L (2017). Examining the human infectious reservoir for *Plasmodium falciparum* malaria in areas of differing transmission intensity. Nat Commun.

[11] Ali D, Brooker SJ, Roschnik N, Witek-McManus S, Verney A, Halliday KE (2015). The high burden of malaria in primary school children in southern Malawi. Am J Trop Med Hyg.

[12] Cohee LM, Peterson I, Buchwald AG, Coalson JE, Valim C, Chilombe M (2022). School-based malaria screening and treatment reduces *Plasmodium falciparum* infection and anemia prevalence in two transmission settings in Malawi. J Infect Dis.

[13] Walldorf JA, Cohee LM, Coalson JE, Bauleni A, Nkanaunena K, Kapito-Tembo A (2015). School-age children are a reservoir of malaria infection in Malawi. PLoS ONE.

[14] Coalson JE, Walldorf JA, Cohee LM, Ismail MD, Mathanga D, Cordy RJ (2016). High prevalence of *Plasmodium falciparum* gametocyte infections in school-age children using molecular detection: patterns and predictors of risk from a cross-sectional study in southern Malawi. Malar J.

[15] Coalson JE, Cohee LM, Buchwald AG, Nyambalo A, Kubale J, Seydel KB (2018). Simulation models predict that school-age children are responsible for most human-to-mosquito *Plasmodium falciparum* transmission in southern Malawi. Malar J.

[16] Willems T, Gymrek M, Highnam G, Mittelman D, Erlich Y (2014). The landscape of human STR variation. Genome Res.

[17] Rosenberg NA, Pritchard JK, Weber JL, Cann HM, Kidd KK, Zhivotovsky LA (2002). Genetic structure of human populations. Science.

[18] Mirghani SE, Nour BY, Bushra SM, Elhassan IM, Snow RW, Noor AM (2010). The spatial-temporal clustering of *Plasmodium falciparum* infection over eleven years in Gezira State. Sudan Malar J.

[19] Smith JL, Auala J, Tambo M, Haindongo E, Katokele S, Uusiku P (2017). Spatial clustering of patent and sub-patent malaria infections in northern Namibia: implications for surveillance and response strategies for elimination. PLoS ONE.

[20] Muirhead Thompson RC (1951). The distribution of anopheline mosquito bites among different age groups; a new factor in malaria epidemiology. BMJ.

[21] Carnevale P, Frézil JL, Bosseno MF, Le Pont F, Lancien J (1978). [The aggressiveness of *Anopheles gambiae* a in relation to the age and sex of the human subjects] (in French). Bull World Health Organ.

[22] Buchwald AG, Walldorf JA, Cohee LM, Coalson JE, Chimbiya N, Bauleni A (2016). Bed net use among school-aged children after a universal bed net campaign in Malawi. Malar J.

[23] Olapeju B, Choiriyyah I, Lynch M, Acosta A, Blaufuss S, Filemyr E (2018). Age and gender trends in insecticide-treated net use in sub-Saharan Africa: a multi-country analysis. Malar J.

[24] Lacroix R, Mukabana WR, Gouagna LC, Koella JC (2005). Malaria infection increases attractiveness of humans to mosquitoes. PLoS Biol.

[25] De Moraes CM, Stanczyk NM, Betz HS, Pulido H, Sim DG, Read AF (2014). Malaria-induced changes in host odors enhance mosquito attraction. Proc Natl Acad Sci USA.

[26] Mbewe RB, Keven JB, Mzilahowa T, Mathanga D, Wilson M, Cohee L (2022). Blood-feeding patterns of *Anopheles* vectors of human malaria in Malawi: implications for malaria transmission and effectiveness of LLIN interventions. Malar J.

[27] ICF International, Ministry of Health (Malawi), National Malaria Control Program (Malawi), National Statistical Office of Malawi. Malawi Malaria Indicator Survey 2014. Fairfax, United States of America: ICF International, 2015.

[28] Cohee LM, Goupeyou-Youmsi J, Seydel KB, Mangani C, Ntenda P, Sixpence A (2022). Understanding the intransigence of malaria in Malawi. Am J Trop Med Hyg.

[29] Coetzee M (2020). Key to the females of afrotropical *Anopheles* mosquitoes (diptera: culicidae). Malar J.

[30] Keven JB, Artzberger G, Gillies ML, Mbewe RB, Walker ED (2020). Probe-based multiplex qPCR identifies blood-meal hosts in *Anopheles* mosquitoes from Papua New Guinea. Parasit Vectors.

[31] Oostdik K, Lenz K, Nye J, Schelling K, Yet D, Bruski S (2014). Developmental validation of the powerplex® fusion system for analysis of casework and reference samples: a 24-locus multiplex for new database standards. Forensic Sci Int Genet.

[32] Findlay I, Matthews P, Quirke P (1998). Multiple genetic diagnoses from single cells using multiplex PCR: reliability and allele dropout. Prenat Diagn.

[33] Kleiber C, Zeileis A (2016). Visualizing count data regressions using rootograms. Am Stat.

[34] Edman JD, Chow-Shaffer E, Clark GG, De Benedictis J, Costero A, Scott TW (2003). Identification of the people from whom engorged *Aedes aegypti* took blood meals in Florida, Puerto Rico, using polymerase chain reaction-based DNA profiling. Am J Trop Med Hyg.

[35] Guelbéogo WM, Gonçalves BP, Grignard L, Bradley J, Serme SS, Hellewell J (2018). Variation in natural exposure to *Anopheles* mosquitoes and its effects on malaria transmission. eLife.

[36] Harrington LC, Fleisher A, Ruiz-Moreno D, Vermeylen F, Wa CV, Poulson RL (2014). Heterogeneous feeding patterns of the dengue vector, *Aedes aegypti*, on individual human hosts in rural Thailand. PLoS Negl Trop Dis.

[37] Paul MR, Grenfell BT, Hoti SL, Ramaiah KD, Bundy DA, Das PK (2001). Quantifying mosquito biting patterns on humans by DNA fingerprinting of bloodmeals. Am J Trop Med Hyg.

[38] McCann RS, Messina JP, MacFarlane DW, Bayoh MN, Gimnig JE, Giorgi E (2017). Explaining variation in adult *Anopheles* indoor resting abundance: the relative effects of larval habitat proximity and insecticide-treated bed net use. Malar J.

[39] Bayoh MN, Walker ED, Kosgei J, Ombok M, Olang GB, Githeko AK (2014). Persistently high estimates of late night, indoor exposure to malaria vectors despite high coverage of insecticide treated nets. Parasit Vectors.

[40] Rodríguez-Rodríguez D, Katusele M, Auwun A, Marem M, Robinson LJ, Laman M (2021). Human behavior, livelihood, and malaria transmission in two sites of Papua New Guinea. J Infect Dis.

[41] Smith DL, Dushoff J, McKenzie FE (2004). The risk of a mosquito-borne infection in a heterogeneous environment. PLoS Biol.

[42] Scott TW, Harrington LC, Yan G, Githeko AK, Fleisher A (2006). DNA profiling of human blood in *Anophelines* from lowland and highland sites in Western Kenya. Am J Trop Med Hyg.

[43] Boreham PFL, Garrett-Jones C (1973). Prevalence of mixed blood meals and double feeding in a malaria vector (*Anopheles sacharovi* Favre). Bull World Health Organ.

[44] Tedrow RE, Zimmerman PA, Abbott KC (2019). Multiple blood feeding: a force multiplier for transmission. Trends Parasitol.

[45] Burkot TR, Graves PM, Paru R, Lagog M (1988). Mixed blood feeding by the malaria vectors in the *Anopheles punctulatus* complex (diptera: culicidae). J Med Entomol.

[46] Chow-Shaffer E, Sina B, Hawley WA, De Benedictis J, Scott TW (2000). Laboratory and field evaluation of polymerase chain reaction-based forensic DNA profiling for use in identification of human blood meal sources of *Aedes aegypti* (diptera: culicidae). J Med Entomol.

[47] Soremekun S, Maxwell C, Zuwakuu M, Chen C, Michael E, Curtis C (2004). Measuring the efficacy of insecticide treated bednets: the use of DNA fingerprinting to increase the accuracy of personal protection estimates in Tanzania. Trop Med Int Health.

[48] Keven JB, Walker ED, Venta PJ (2019). A microsatellite multiplex assay for profiling pig DNA in mosquito bloodmeals. J Med Entomol.

[49] Zhou Z, Mitchell RM, Kariuki S, Odero C, Otieno P, Otieno K (2016). Assessment of submicroscopic infections and gametocyte carriage of *Plasmodium falciparum* during peak malaria transmission season in a community-based cross-sectional survey in western Kenya, 2012. Malar J.

[50] Andolina C, Rek JC, Briggs J, Okoth J, Musiime A, Ramjith J (2021). Sources of persistent malaria transmission in a setting with effective malaria control in eastern Uganda: a longitudinal, observational cohort study. Lancet Infect Dis.

[51] Ouédraogo AL, Gonçalves BP, Gnémé A, Wenger EA, Guelbeogo MW, Ouédraogo A (2016). Dynamics of the human infectious reservoir for malaria determined by mosquito feeding assays and ultrasensitive malaria diagnosis in Burkina Faso. J Infect Dis.

