# Supplementary material for: Genotyping of Anopheles mosquito blood meals reveals nonrandom human host selection: implications for human-to-mosquito Plasmodium falciparum transmission
Source: Malar J. 2023 Apr 7;22:115. doi: 10.1186/s12936-023-04541-2 (PMC10080529; doi:10.1186/s12936-023-04541-2)
Supplement: Supplementary file 1 — Additional file 1: Figure S1. Example of output for visualization of allelic sizes on each loci for creating genetic profiles of each individual sample. [file 12936_2023_4541_MOESM1_ESM.docx]

**Supplementary Figure.**

Figure S1. Example of output for visualization of allelic sizes on each loci for creating genetic profiles of each individual sample.
