# Supplementary material for: Genotyping of Anopheles mosquito blood meals reveals nonrandom human host selection: implications for human-to-mosquito Plasmodium falciparum transmission
Source: Malar J. 2023 Apr 7;22:115. doi: 10.1186/s12936-023-04541-2 (PMC10080529; doi:10.1186/s12936-023-04541-2)
Supplement: Supplementary file 2 — Additional file 2: Table S1. Percent of loci matched, pairwise testing matches and probability of having similar genetic makeup among 190 sampled human individuals (29,161 pairwise comparisons) from 46 different households. [file 12936_2023_4541_MOESM2_ESM.docx]

Table S1. Percent of loci matched, pairwise testing matches and probability of having similar genetic makeup among 190 sampled human individuals (29,161 pairwise comparisons) from 46 different households.

| **Percentage matched (%)** | **No. of pairwise matches** | **Probability of a pairwise**  **match** |
| --- | --- | --- |
| **0** | 2612 | 0.090 |
| **4** | 5161 | 0.177 |
| **8** | 7397 | 0.254 |
| **12** | 6470 | 0.222 |
| **17** | 3813 | 0.131 |
| **21** | 1989 | 0.068 |
| **25** | 832 | 0.029 |
| **29** | 376 | 0.013 |
| **33** | 208 | 0.007 |
| **38** | 133 | 0.005 |
| **42** | 69 | 0.002 |
| **46** | 47 | 0.00161 |
| **50** | 14 | 0.00048 |
| **54** | 12 | 0.00041 |
| **58** | 14 | 0.00048 |
| **62** | 8 | 0.00027 |
| **67** | 2 | 0.00007 |
| **71** | 1 | 0.00003 |
| **75** | 1 | 0.00003 |
| **79** | 2 | 0.00007 |
