# Supplementary material for: Genotyping of Anopheles mosquito blood meals reveals nonrandom human host selection: implications for human-to-mosquito Plasmodium falciparum transmission
Source: Malar J. 2023 Apr 7;22:115. doi: 10.1186/s12936-023-04541-2 (PMC10080529; doi:10.1186/s12936-023-04541-2)
Supplement: Supplementary file 3 — Additional file 3: Table S2. Demographic age groups separated by sex showing their P. falciparum infection proportions. [file 12936_2023_4541_MOESM3_ESM.docx]

**Table S2. Demographic age groups separated by sex showing their *P. falciparum* infection proportions.**

|  | | Proportion of infections | |
| --- | --- | --- | --- |
| Human host | | **Infected**  **blood meal*** | **Uninfected blood meal** |
| ≤5y | Male | 1 (0.20) | 4 (0.80) |
|  | Female | 7 (0.78) | 2 (0.22) |
| 6-15y | Male | 9 (0.41) | 13 (0.59) |
|  | Female | 17 (0.43) | 23 (0.57) |
| 16-30y | Male | 0 (0) | 19(100) |
|  | Female | 3 (0.43) | 4 (0.57) |
| 31-75y | Male | 10 (0.16) | 53 (0.84) |
|  | Female | 2 (0.13) | 13 (0.87) |

The SAC had the highest number of infections, however, in relation to their proportions in the population, females of ≤5 years old, males and females of 6-15 years old and females of 16-30 years old, all had high infection rates. Data were too sparse for statistical inferences.
